# New Approaches to Stretched Film Sample Alignment and Data Collection for Vibrational Linear Dichroism

**DOI:** 10.1021/acsomega.3c05774

**Published:** 2023-09-28

**Authors:** Paul Wormell, Pavel Michal, Adam Scott, Koushik Venkatesan, Kausala Mylvaganam, Tobias von Arx, Junya Kitamura, Jun Koshoubu, Alison Rodger

**Affiliations:** † School of Science, Western Sydney University, Locked Bag 1797, Penrith, New South Wales 2751, Australia; ‡ Department of Optics, Palacký University Olomouc, 17. Listopadu 12, Olomouc 77146, Czech Republic; § School of Natural Sciences, Macquarie University, Sydney, New South Wales 2109, Australia; ∥ JASCO International Co., Ltd, Hachioji, Tokyo 192-0046, Japan; ⊥ JASCO Corporation, Hachioji, Tokyo 192-8537, Japan

## Abstract

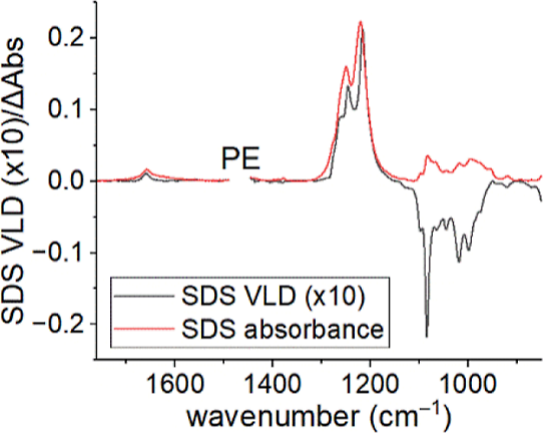

Rapid measurements of vibrational linear dichroism (VLD) infrared spectra are shown to be possible by using stretched polymer films and an extension of existing instrumentation designed for vibrational circular dichroism spectroscopy. Earlier techniques can be extended using additional inexpensive polymer substrates to record good-quality VLD spectra of a significantly wider range of compounds with comparatively short sample-preparation times. The polymer substrates used, polyethylene and polytetrafluoroethylene, are commonly available and inexpensive, and samples are more easily prepared than that for many earlier stretched-film and crystal studies. Data are presented for neutral hydrophobic organic molecules on hydrophobic films including acridine, anthracene, fluorene, and recently synthesized *S*-(4-((4-cyanophenyl)ethynyl)phenyl)ethanethioate. We extend the approach to polar or ionic species, including 2,2′-bipyridine, 1,10-phenanthroline, and sodium dodecyl sulfate, by oxidizing polyethylene films to change their wetting properties. The combination of new instrumentation and modified sample preparation methods is useful in basic spectroscopy for untangling and assigning complicated infrared spectra. Nevertheless, it is not a panacea as surface-adsorbed molecules are often not monodispersed, and higher analyte concentrations can lead to aggregation and resonance phenomena that have previously been observed for infrared spectra on surfaces. These effects can be assessed by varying the sample concentration. The focus of this paper is experimental, and detailed analysis of most of the spectra lies outside its scope, including some well-studied compounds such as acridine and anthracene that allow comparisons with earlier research.

## Introduction

Mid-infrared (IR) absorption spectroscopy (4000–400 cm^–1^) is a valuable fast characterization tool for molecular identification, structure determination, and sometimes quantitation of components. IR spectra typically provide rich spectroscopic and structural information about molecules undergoing vibrational transitions. Applications are diverse, including nanomaterials,^1^ medicine,^2^ biological science,^3^ forensic science,^4^ environmental analysis,^5^ and soils.^6^

Polarization-dependent IR absorption can provide additional spectroscopic information. One common technique is vibrational circular dichroism (VCD),^7–9^ which is based on the different interactions of left and right circularly polarized IR radiation with chiral molecules. The unique sign pattern and relative intensities of vibrational bands can reveal the absolute stereochemistry and spatial arrangement of chiral molecules in the conformational space.^10^ The VCD spectra can be recorded rapidly in favorable cases such as pure liquid α-pinene, but long data collection times are often required for less concentrated samples.^8,11^ Another method is vibrational linear dichroism (VLD) of oriented samples
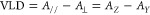
which measures the difference in IR absorbance of radiation polarized parallel (*Z*) and perpendicular (*Y*) to an orientation direction. In this axis system, the radiation propagates along *X*. Data analysis often proceeds via the reduced LD, LD^*r*^, which for the simple case of uniaxial alignment^12,13^ is given by
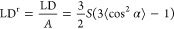
where α is the angle between the molecule’s orientation axis (see below) and the polarization of the transition of interest, the angular brackets denote average over the population of molecules present (note this is the average of the square of the cosine rather than the average of the angle), and *S* is the orientation parameter (0 for unoriented samples, 1 for perfectly oriented samples). The signals are thus modulated by the angle the transition moment makes to the molecular orientation direction, as well as by the degree of orientation. This gives an extra dimension to the spectrum, enabling us to determine transition polarizations and/or orientations of vibrational chromophores relative to an orientation axis.

Earlier VLD experiments using pure crystals,^14,15^ stretched films,^12,16–31^ and nematic crystals^25,32–34^ were performed by inserting a polarizer in the IR beam and then either rotating the polarizer or the film. The former risks different intensity beams, and the latter risks the beam passing through different parts of the sample, both of which distort the VLD spectrum. UV–visible LD measurements have long been possible using spectrometers that allow the difference and sum of the two polarizations to be measured simply and rapidly without moving the sample or rotating any optical components.^13^ Similar approaches and instrumentation have also been used for earlier polarized IR measurements, including dynamic IR linear dichroism spectroscopy^35,36^ and polarization-modulation IR reflection absorption spectroscopy.^37,38^ We have extended our own UV–visible approaches to mid-IR wavelengths using a conversion kit to adapt an existing Jasco FVS-6000 VCD spectrometer for VLD measurements. Thus, it is timely to consider how VLD can be further developed as a readily accessible molecular characterization technique, where a key to future success is to have simple sample orientation methods appropriate for different sample types.

The film LD work noted above was largely undertaken using polyethylene (PE) films, either cast by melting PE pellets or powder, or commercially available films of various thicknesses, sometimes in multiple layers, or sections from PE bottles.^21,22^ The films were stretched, typically by factors of 3–6× to orient them. Analytes were introduced by soaking, usually in a chlorinated solvent,^16,39–42^ or from a neat liquid,^18^ or by sublimation,^17^ either before or after stretching, depending on the study. In general, the longest molecular axis partially aligns along the film stretch direction *Z*.^12,40^ An alternative nonpolar film that has been used with some success is polytetrafluoroethylene (PTFE) on glass or silicon.^43,44^ Stretched poly(fluorinated ethylene-propylene) films have been used to obtain polarized UV–visible absorption spectra of radical ions of some azanaphthalenes and biphenyls.^45^ For polar analytes, polymers such as poly(vinyl alcohol) (polymerized with the analyte and left to dry for days) have previously been adopted,^12,13,46^ although PE has also been a successful host for some compounds.^18,26^ These approaches to sample orientation, while successful, are often time-consuming, taking several hours or days to complete. We therefore considered how to extend our work on simple UV–visible LD sample presentation to the IR. We have found that commercial PE plastic bags orient nonpolar molecules well with an optimal stretch factor of ∼2–3 (2 is optimal for Glad PE sandwich bags, our preferred PE substrate).^47^ Razmkhah et al.^47^ extended the simplicity of commercial PE to polar molecules by oxidizing Glad PE sandwich bags in a plasma asher to oxidize the surface at a very low level which proved sufficient to change to the way water and hydrophilic analytes interacted with the PE surface, spreading rather than sitting above the surface as a high-curvature droplet. In this work, we have taken the achievements of film orientation developed for IR and UV–visible spectroscopy and enhanced them to provide a suite of options that can readily be implemented for VLD in the laboratory including PE sandwich bags and a textured PE wrapping film (Glad Press’n Seal film) in their original and oxidized forms as well as various commercial PTFE tapes. As for UV–visible LD, this has allowed us to extend the range of analytes to more polar compounds including 2,2′-bipyridine and 1,10-phenanthroline and the ionic compound sodium dodecyl sulfate (SDS).

## Methods

### Analytes

**Figure 1:**
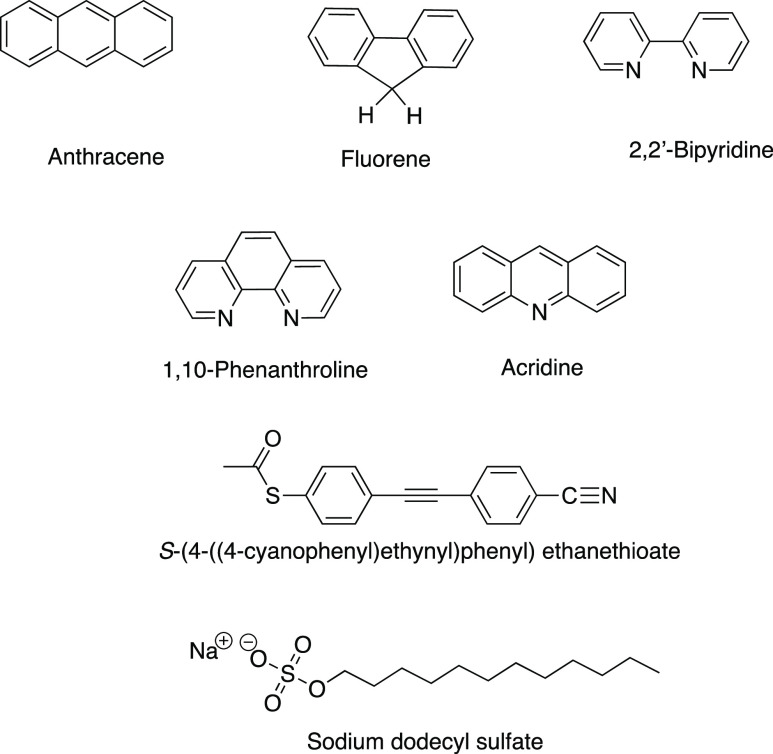
Molecular structures for analytes used in this work.

The following compounds were used for VLD spectroscopy in this work: acridine (Sigma-Aldrich, 97%); anthracene (BDH, recrystallized from ethanol); fluorene (BDH, recrystallized from ethanol); *S*-(4-((4-Cyanophenyl)ethynyl)phenyl)ethanethioate (see Figure 1; synthesized as set out below); 2,2′-bipyridine (BDH, zone-refined, 70 passes); 1,10-phenanthroline (Aldrich, recrystallized from heptane); and SDS [Sigma, dust-free pellets, suitable for electrophoresis for molecular biology, ≥99.0% (by GC)]. The solvents used for sample preparation were chloroform (Sigma-Aldrich, ≥99.5%, contains 100–200 ppm amylenes as a stabilizer), methanol (Sigma-Aldrich), and water (Direct-Q RO, 18.2 MΩ).

Structures of the analytes are shown in Figure 1.

*S*-(4-((4-Cyanophenyl)ethynyl)phenyl)ethanethioate was synthesized starting from cyano-(ethynyl)benzene and iodobenzene-4-thioacetate under Sonogashira coupling conditions in 57% yield. Iodobenzene-4-thioacetate^48^ and cyano-4-(ethynyl)benzene^49^ were synthesized following previously reported procedures.^48,49^ See the Supporting Information for details.

### PE and PTFE Films

PE films were prepared from Glad Snap Lock sandwich bags, unstretched thickness of 0.05 mm, and Glad Press’n Seal film, unstretched thickness of 0.025 mm. Commercial poly(tetrafluoroethylene) (PTFE) tapes are available in a range of colors and sizes. In this study, the PTFE films were prepared from Kinetic standard white PTFE thread seal tape, 12 mm width (typical of many other commercial tapes), unstretched thickness 0.1 mm, and Unasco white PTFE thread seal tape, 25 mm width, unstretched thickness 0.076 mm (nominal), obtained from ATOM, Wetherill Park, NSW, Australia. All thickness and width measurements are as quoted by the manufacturers. The tape is white and opaque, making it unsuitable for linear dichroism measurements at UV and visible wavelengths. However, scattering decreases with the increasing wavelength, and the stretched tape was sufficiently transparent for us to record useable mid-IR absorption and LD spectra. For most VLD spectra, we used two pieces of thin white Kinetic standard PTFE thread seal tape, 12 mm width, which gave a smaller background spectrum and hence was preferred over thicker tapes.

### Surface-Oxidized PE (PE^OX^) and PTFE Films

Strips of the PE film (2.5 × 4 cm) were placed in a Diener Zepto Plasma Asher connected to an oxygen gas supply for 1 min at 50 W power setting and a pressure of 0.2–0.5 mbar. This level of treatment produced films with adequate wetting behavior with aqueous solutions. This can be further enhanced by longer oxidation periods. PTFE films can also be oxidized in this way, although they can become sticky and brittle and were not needed for the sample compounds used in the current study.

### Film Stretching

**Figure 2:**
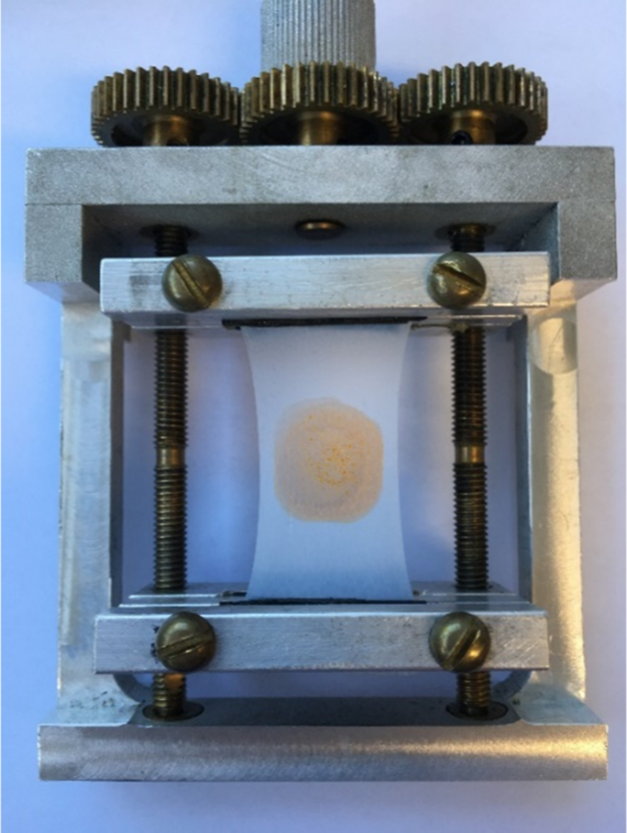
Film stretcher with a reverse screw thread showing a patch of tetracene on the 2× stretched Glad Press’n Seal PE film.

All VLD spectra were collected on films mounted in a mechanical stretcher with a reverse thread screw (Figure 2).^47^ This was designed to stretch the samples uniformly across the width of the film with stretching from both directions so that a sample loaded in the center of the film remained in the center. PE and PE^OX^ films of size 2.5 × 4 cm, or a single length of 25 mm PTFE tape, or two lengths of 12 mm PTFE tape, slightly overlapping, were placed between the jaws of the stretcher. Razmkhah et al.^47^ found that the maximum LD was obtained with a stretch factor of about 1.8 and a maximum LD^r^ with a factor of about 2 with PE. Accordingly, our films were stretched by a factor of 2 (PE and PE^OX^) or 1.5–1.9 (PTFE). The PTFE stretch depended on the sample and was performed to the point at which the film started to form voids, and the fibrillar structure was clearly apparent. All operations were carried out at room temperature (∼20 °C).

### Sample Loading onto Films

Although the commercial PE films used in this study are much thinner than many of the films used in earlier studies,^20,32^ the sensitivity of the adapted spectrometer allows useable spectra to be recorded of compounds absorbed into films and adsorbed on the surface.

To absorb analytes into stretched PE films, we immersed unstretched films in concentrated analyte solutions in chloroform (typically 20 mg/mL) for 20–60 min. The films were allowed to dry, mounted in the stretcher, and stretched by a factor of 2.0. Following Thulstrup et al.,^40^ Radziszewski & Michl,^20^ and Wormell & Lacey,^41^ the surface was then wiped with paper tissue soaked in methanol to remove surface molecules. Absorption and VLD (see below) spectra were recorded. The stretched film was soaked for about 30 s in paper tissue with chloroform to extract the analyte enabling a polymer baseline spectrum to be collected (see the Supporting Information for details of baseline correction). Alternatively, baseline spectra of the films were collected before adding the sample but after swelling the film with the intended solvent and then allowing it to evaporate. Both approaches gave similar results as soaking was a very effective way of extracting the analyte.

To adsorb analytes onto a film surface, they were dissolved in an appropriate solvent at concentrations ranging from 1 to 20 mg/mL. 20 μL aliquots of the solution were placed on the film and allowed to spread and dry, usually without any interventions except for aqueous solutions, which were blown dry in a stream of nitrogen. Chloroform solutions typically dried within 1 min, but aqueous solutions took from a few minutes to tens of minutes to dry, depending on the solute, sample volume, and concentration. Low concentrations typically gave an even spread of solute. Repeated aliquots usually produced layering, leading to “tide marks” or “coffee rings”,^50^ and shiny polycrystalline regions with associated complexity in the VLD spectra (see below). Sample patches for all samples in this study were larger than the IR beam diameter of 7.1 mm.

### VLD Measurements on Stretched Films

Absorption and LD spectra were recorded using a Jasco FVS-6000 VCD spectrometer with a mercury cadmium telluride detector, adapted for LD measurements in the 850–2000 and 2000–3200 cm^–1^ ranges. The conversion kit was installed by the manufacturer with the option of recording either VCD or VLD spectra. For survey spectra in the 850–2000 cm^–1^ range, a spectral bandwidth of 4 cm^–1^ was satisfactory. However, a 1 cm^–1^ bandwidth gave sharper bands and a clearer indication of any water-vapor bands that might be affecting the baseline. Since it was hard to discern useable bands in the 2000–3200 cm^–1^ range for the analytes that we studied, we have only reported a small number of spectra in this range (see Figure 6). Spectra were recorded with nitrogen purging of the sample compartment to minimize the intensity of water bands. 5 L min^–1^ was typically used for initial purging, with at least 3 L min^–1^ required to keep the concentration of water vapor low. The flow rate was adjusted during data collection based on water vapor peaks centered on ∼1600 cm^–1^. Typically 500 scans were recorded, taking 13 min per spectrum, but a smaller number of scans would have given acceptable spectra for many of our samples. The data acquisition and baseline subtraction were performed using the Spectra Manager software version 2.07.02, as outlined in the Supporting Information.

**Figure 3:**
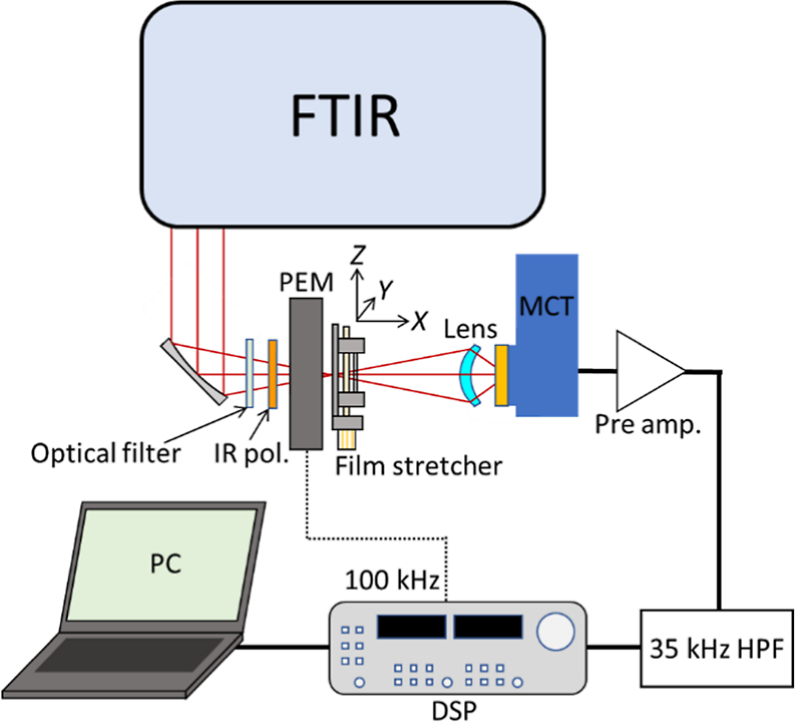
Schematic diagram of the Jasco FVS-6000 VCD spectrometer in the VLD mode.

As with typical implementation of electronic LD (ELD),^13^ the VLD instrument is based on a VCD instrument in this case producing alternating beams of parallel (vertical in our instrument; *Z*) and perpendicular (horizontal; *Y*) polarization. Figure 3 shows a schematic diagram of the VLD measurement system with a sample stretcher. The stretching direction coincides with the *Z* direction. The IR radiation (propagating in the *X* direction) from the FTIR spectrometer is converted to linearly polarized IR by a photoelastic modulator (PEM100, Hinds, Hillsboro, OR) operating in a half-wave plate mode with a modulation frequency of 100 kHz. The measured signal was locked-in detection synchronized with the modulation frequency of the PEM by the built-in digital signal processing of the FVS-6000 VCD spectrometer including a 35 kHz high-pass filter and a digital signal processor. This allows the VLD signal of the difference between the parallel (*Z*) and perpendicular (*Y*) orientations to be measured.

We oriented our films so that the stretch direction was vertical, as shown in Figure 3. PE and PE^OX^ films are effectively opaque from 1450 to 1480 cm^–1^. The corresponding opaque region for PTFE is 1130–1260 cm^–1^ which is wider than the PE exclusion zone but completely complementary to it (see Figure SI1 in the Supporting Information). Thus, a combination of films allowed spectra to be recorded throughout the spectrometer range when this was desirable, without needing to use perdeuterated PE as in earlier studies.^20,51^

Simple subtraction of polymer baseline spectra often did not produce analyte spectra with perfectly flat baselines, especially at the sensitivities that were possible by using this spectrometer. Deviations were attributable to slight mismatches in sample placement and sinusoidal features, discussed below, arising from interference fringes caused by the interaction between the IR beam and the thin-polymer films. Baselines were therefore corrected using the Jasco Spectra Manager software version 2.07.02 (see the Supporting Information for details). This was swift and straightforward when the sharpness of the bands was sufficient to make the location of the baseline clear. The baseline correction was more complicated for broader absorption spectra; e.g. from large biomolecules, where the correction relied on the comparison of replicate spectra (to account for residual interference fringes) and surface abrasion of the films to minimize the size of the fringes. This typically took up to 30 min when spectra were broad and quantitation was required. In such cases, replicate spectra may be required to ensure reproducibility. Any weak residual water–vapor rovibrational bands were minimized by adding or subtracting small multiples of a standard water–vapor spectrum (see the Supporting Information).

### Vibrational Computations

An optimized molecular geometry and harmonic vibrational frequencies and transition moments of *S*-(4-((4-cyanophenyl)ethynyl)phenyl)ethanethioate were obtained by density functional theory calculations using the Gaussian 16 Revision A.03 program.^52^ The B3LYP hybrid functional,^53,54^ including GD3 empirical dispersion correction, was used in combination with the 6-311++G(d,p) basis set, as in our earlier successful studies.^55,56^

## Results and Discussion

The results reported in this work illustrate the quality of VLD data that can be collected with an instrument adapted from one designed to measure the much smaller signals of VCD and with commercial polymer films chosen for different types of analyte features (nonpolar vs polar, PE vs PTFE). Although IR molar extinction coefficients are typically lower by a factor of 100–1000 compared with the UV, we were able to use similar analyte concentrations as for ELD due to the sensitivity of the instrument and the lack of scattering in the spectra. Examples of nonpolar (absorbed and adsorbed) as well as polar (adsorbed) molecules are given.

### PE and PTFE Spectra

PE films have relatively narrow absorption regions between 680 and 720 cm^–1^ and 1450 and 1480 cm^–1^ with negative LD signals (see Figure SI1 in the Supporting Information). In addition, there are weak, broad absorption bands centered on 1372, 1443, 1490, and 1731 cm^–1^ and weak, sharp bands at 1051 and 1176 cm^–1^. PTFE contains strong broad bands with negative LD values centered on 1150 and 1200 cm^–1^. The uniform surface of sandwich-bag PE resulted in strong sinusoidal features caused by interference effects from the thin homogeneous uniform PE.^57^ Following the example of Radziszewski and Michl,^20^ abrasion of the surface with commercial grades of abrasive paper reduced the interference fringes but usually did not completely suppress them. A range of grit sizes was tried, ranging from 100- to 1200-grit, with the finer grades giving less risk of cutting through the films. A significant improvement was found when we discovered that the textured Glad Press’n Seal (PE^PnS^) multipurpose sealing wrap and its oxidized form gave weak or even no interference fringes (see Figure SI1 in the Supporting Information). We did not see any difference in the IR or VLD spectra of the oxidized and unoxidized PE or PE^PnS^ films that could be attributed to oxidation. We used PTFE where samples penetrated this film effectively or where its spectral window relative to that of PE was convenient. Its less regular surface meant that interference fringes were not seen. The PE, PE^PnS^, and PTFE orientation parameters were typically *S* ∼ 0.25, ∼ 0.5, and ∼0.1, respectively, assuming α = 90°. Surface oxidation of the PE and PE^PnS^ films did not affect the values of *S*.

### Absorbed Non-Polar Analytes—PE

**Figure 4:**
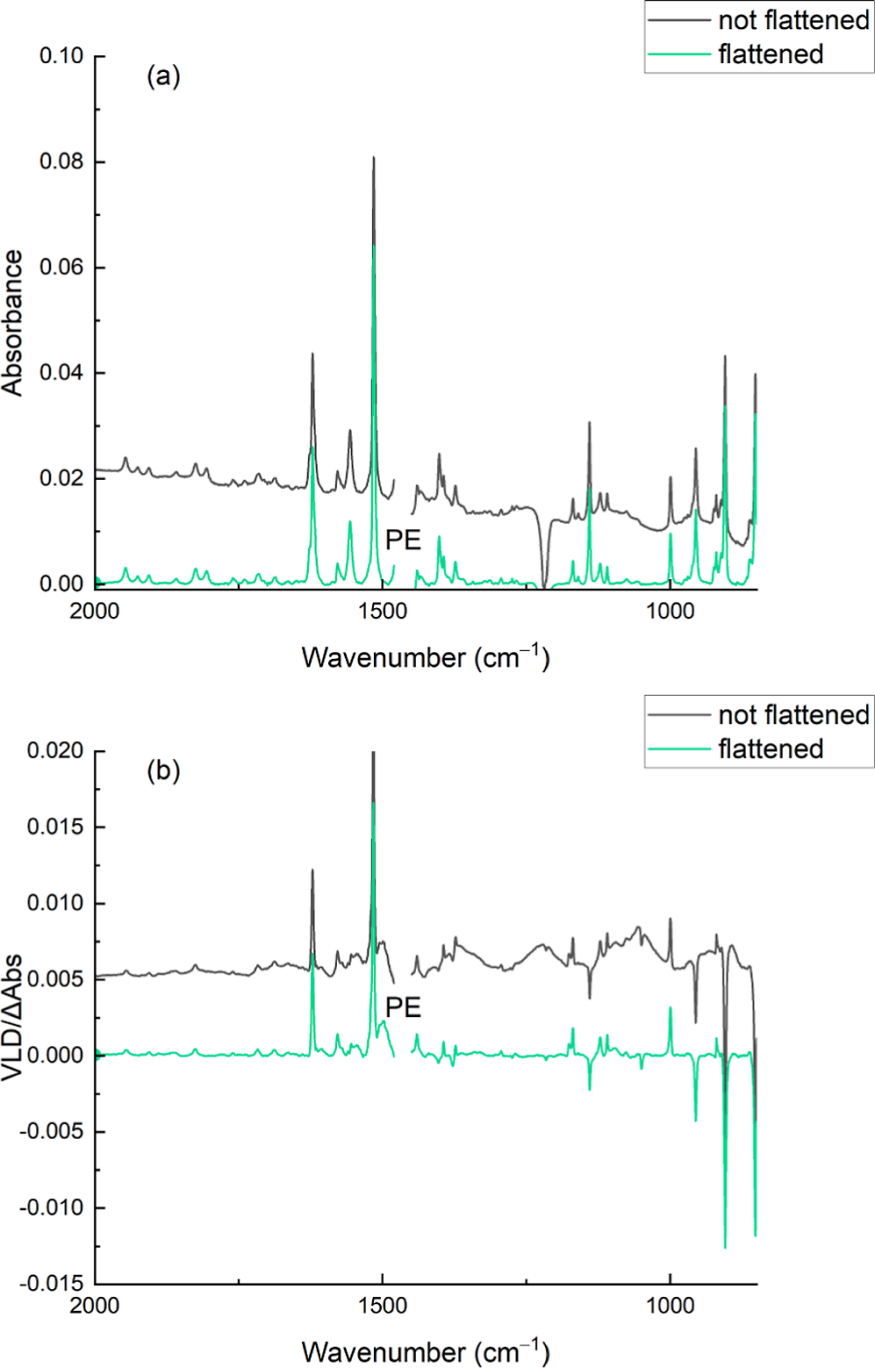
Absorption (a) and VLD (b) spectra of acridine absorbed into a 2× stretched Glad Snap Lock PE film. The PE background spectra have been subtracted (both spectra), and the baseline has been flattened and residual PE bands removed (green spectrum). The spectra are vertically displaced for clarity. Data are available in Supporting Information.

**Figure 5:**
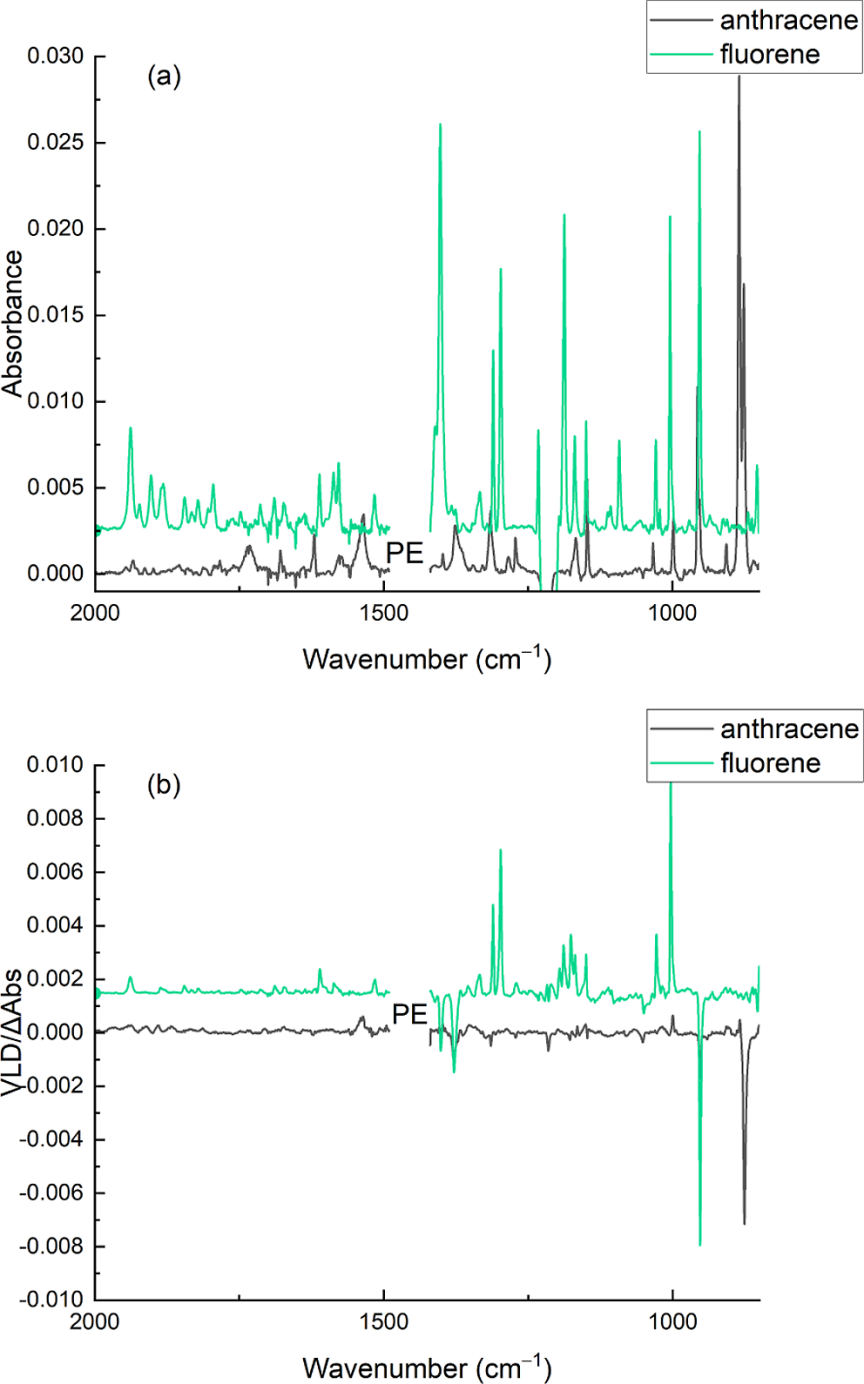
Absorption (a) and VLD (b) spectra of fluorene and anthracene absorbed into a 2× stretched Glad Snap Lock PE film. The PE background spectra have been subtracted, baselines have been flattened, and residual PE bands have been removed. The spectra are vertically displaced for clarity.

Acridine and fluorene are the best examples in the literature of polarized IR spectra for molecules incorporated into PE. Published spectra and tables of data are available from 100 to 3200 cm^–1^ for acridine^19^ and from 400 to 2000 and 2960 to 3140 cm^–1^ for fluorene,^32^ determined from pairs of spectra collected with light polarized parallel and perpendicular, respectively, to the direction of stretch. A table of data is also available for anthracene.^19^Figure 4 shows our IR absorption and VLD spectra of acridine absorbed into PE from chloroform. The baseline-corrected spectrum and the corresponding baseline-flattened spectrum with residual PE bands removed are shown overlaid to indicate the extent of processing undertaken. The baseline flattening process assumes that the absorbance is zero between bands. As an example, band wavenumbers, reduced LD (LD^r^), and calculated orientation parameters (*S*) of VLD spectra of acridine absorbed in ×2 stretched Snap-Lock and Press’n Seal PE films are shown in Table SI1 of the Supporting Information, with literature wavenumbers and band polarizations for comparison.^19^Figure 5 shows the baseline-flattened IR absorption and VLD spectra of fluorene and anthracene absorbed into the stretched PE film. The apparent negative absorption bands centered at 1218 cm^–1^ are artifacts arising from an unknown contaminant introduced during chloroform soaking of the PE film.

In this study, we have found that stretched commercial PE films are suitable for measuring IR band wavenumbers, VLD signs, and approximate relative band intensities and LD^r^ values for analytes that dissolve in PE. Our spectra are consistent with the reference spectra, although showing small intensity differences between samples and missing some of the weaker bands previously observed, as our polymer films were relatively thin. Accordingly, this approach can be used as a swift and straightforward way of generating wavenumber and VLD data for use in assigning molecular vibrations, along the lines of earlier studies that typically used thicker films, including those that were custom-made. The accuracy and precision of our measured band absorbances and VLD values depend on signal intensity and the extent and reliability of baseline flattening. This in turn depends on the size of the interference fringes, if any, and the sharpness of the absorption and VLD bands. Flattening is more reliable when bands are sharp and separate. Difficulties in flattening arise for broad bands with significant interference fringes, although this was not a problem for the analytes reported here. In this case, comparison of repeat spectra can help with identifying the baseline. Reliable relative band intensities would require careful attention to reproducibility, baseline flattening, and minimization of interference fringes for the particular analyte/polymer combination being studied.

In general, spectra were of comparable intensities when collected using PE and PE^PnS^ (data not shown), although there was always some spectrum-to-spectrum variation. Typically, the spectra were weaker in stretched PE^PnS^, correlating with the films being thinner. Analyte orientation parameters for PE are typically between 0.1 and 0.2, compared to the orientation factor of 0.25 for the polymer itself (see Figure SI1 in the Supporting Information) reflecting the orientation distribution of the analytes around the oriented polymers. There was more sample-to-sample variation for Glad Press’n Seal PE (film *S* ∼ 0.5) than that for sandwich bag PE, with values of *S* clustered around 0.10 (acridine), 0.10–0.40 (fluorene), and 0.10–0.20 (anthracene). Surface oxidation did not affect the spectra observed for nonpolar analytes. However, note that our approach does not always work. For example, azulene was well-absorbed by the PE films but rapidly evaporated in the nitrogen-purged sample compartment. The thicker films of previous work would be more suitable for such samples.^20^

With our current approach, the polarization information is obtained swiftly and presented as a single spectrum from which LD^r^ values and orientation parameters are readily calculated. A plot of LD^r^ versus wavenumber fluctuates wildly, mainly as a result of dividing two near-zero quantities for most data points, and the useable LD^r^ values are only in the middle of the absorption bands (Figure 5, green and black spectra). In the literature, LD^r^ values have always been quoted not plotted for this reason.

In our previous work on ELD of aromatic molecules, we noted significant variations in the spectra as a function of loading due to electronic coupling between stacked molecules. Loading dependence is less apparent in the IR than the UV, though there was some variability in LD^r^ values and orientation parameters, *S*, within and between spectra.

### Adsorbed Nonpolar Analytes—PTFE Data

**Figure 6:**
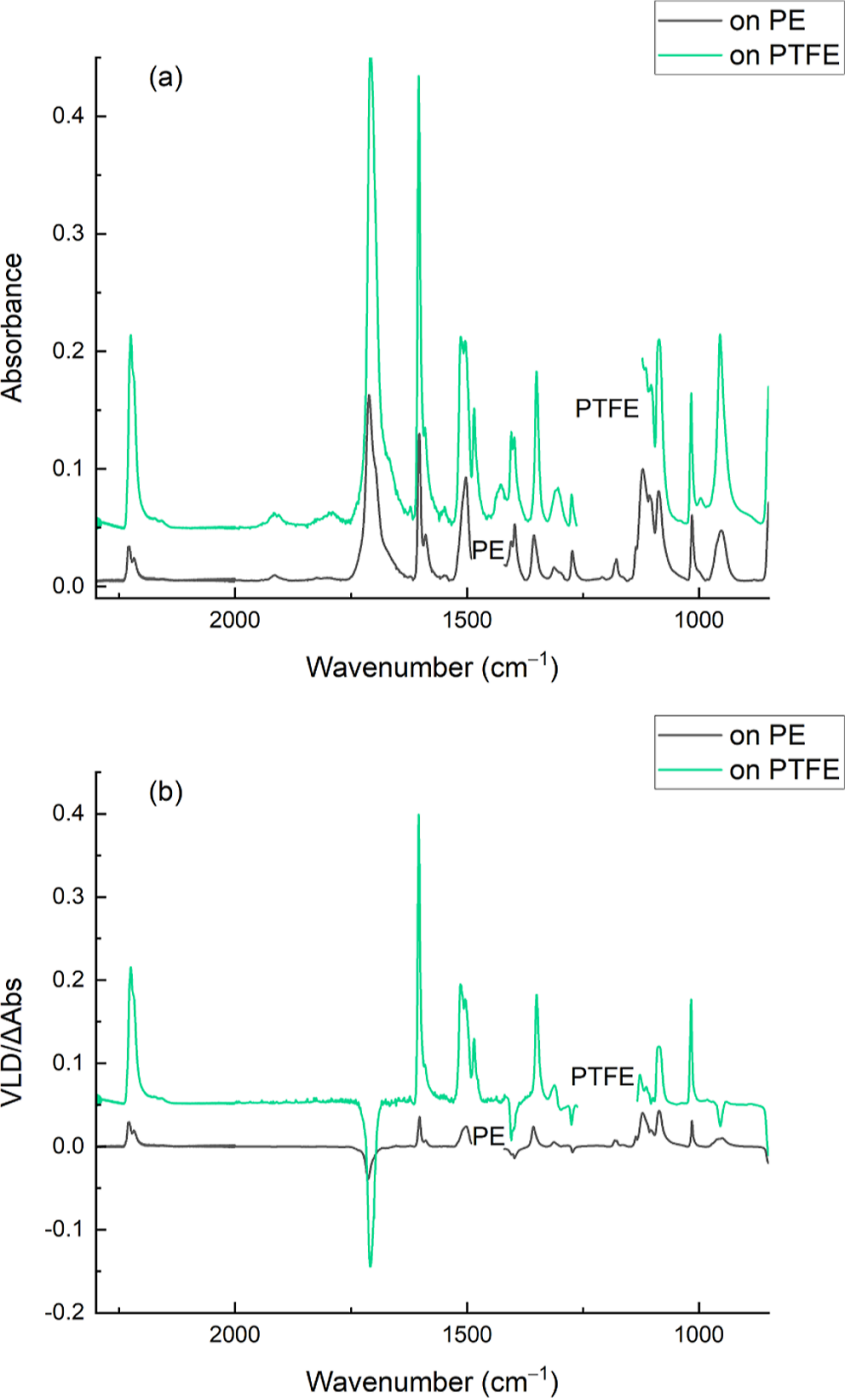
Absorption (a) and VLD (b) spectra of *S*-(4-((4-cyanophenyl)ethynyl)phenyl)ethanethioate adsorbed onto 1.85× stretched PTFE film and 2× stretched PE^PnS,OX^ film. Polymer spectra have been subtracted, baselines have been flattened, including removal of interference fringes, and the residual polymer bands have been removed. The spectrum on PE^PnS,OX^ has been displaced vertically for clarity.

The quality of data obtainable with PTFE is illustrated in Figure 6 for *S*-(4-((4-cyanophenyl)ethynyl)phenyl)ethanethioate where a strong VLD spectrum (LD ∼ 0.35 and LD^r^ ∼ 0.89, *S* ∼ 0.3) results from depositing the analyte from chloroform solution onto a stretched PTFE film. A useable spectrum was obtained within 30 min. This illustrates the ease with which VLD measurements can be obtained for a recently synthesized compound. The spectrum recorded on stretched PE^PnS^ is also shown in Figure 6 for comparison. The same sample concentrations and volumes were used for the two spectra, and the sample patches were of a similar size. The spectrum on PE is weaker than the spectrum on PTFE as the sample solution does not penetrate the film and visibly produces unoriented polycrystalline deposits at higher sample concentrations. For this molecule, the VLD spectrum assists us in assigning the absorption bands by using experimental and computed band wavenumbers and polarizations. We note, however, that this is intended to show how VLD data can be readily obtained for a new compound rather than give a detailed analysis of the molecular vibrations of this and related compounds, which lies outside the scope of the present paper. However, for illustrative purposes, we carried out some preliminary gas-phase B3LYP/6311 + G(d,p) computations of the vibrational frequencies and polarization directions to evaluate the VLD signs of some major bands. A comparison of theory and experiment shows that the spectrum contains some strong vibrational bands that can be readily attributed to the functional groups in the molecule. For example, the experimental doublet at 2219/2225 cm^–1^ (see Figure 6) was successfully reproduced by our preliminary computations and revealed different combinations of C≡C and C≡N stretching modes. These lie in the higher (2000–3200 cm^–1^) wavenumber range of the spectrometer and have the positive LD expected for transitions polarized along the molecular axis. Other examples of prominent bands include a short-axis polarized S–C=O deformation with negative LD at 1707 cm^–1^, and a long-axis polarized mode chiefly involving C–C stretches in the benzene rings, with positive LD, at 1604 cm^–1^.

### Adsorbed Polar Analytes—PE^OX^, PE^PnS,OX^, and PTFE

Molecules ranging from small polar heterocycles to large polar and ionic species, including surfactants, are not readily incorporated into PE films. Following the earlier UV–visible work with PE^OX^ of Razmkhah et al.,^47^ we found PE^OX^ and PE^PnS,OX^ were effective substrates for orienting polar samples for VLD. We also found that nonpolar, stretched PTFE can produce useful VLD spectra of surface-adsorbed polar molecules if access to an analyte spectrum at wavenumbers where PE absorbs is required. However, we used PE^OX^ and PE^PnS,OX^ where possible as aqueous solutions on hydrophobic PTFE surfaces bear some resemblance to mercury droplets on a sheet of glass. Accordingly, spreading and drying these solutions is much harder than samples on oxidized PE films. As stated above, oxidized PTFE films can become sticky and brittle and were not needed for the sample compounds used in the current study, so we did not attempt to optimize the oxidation parameters.

**Figure 7:**
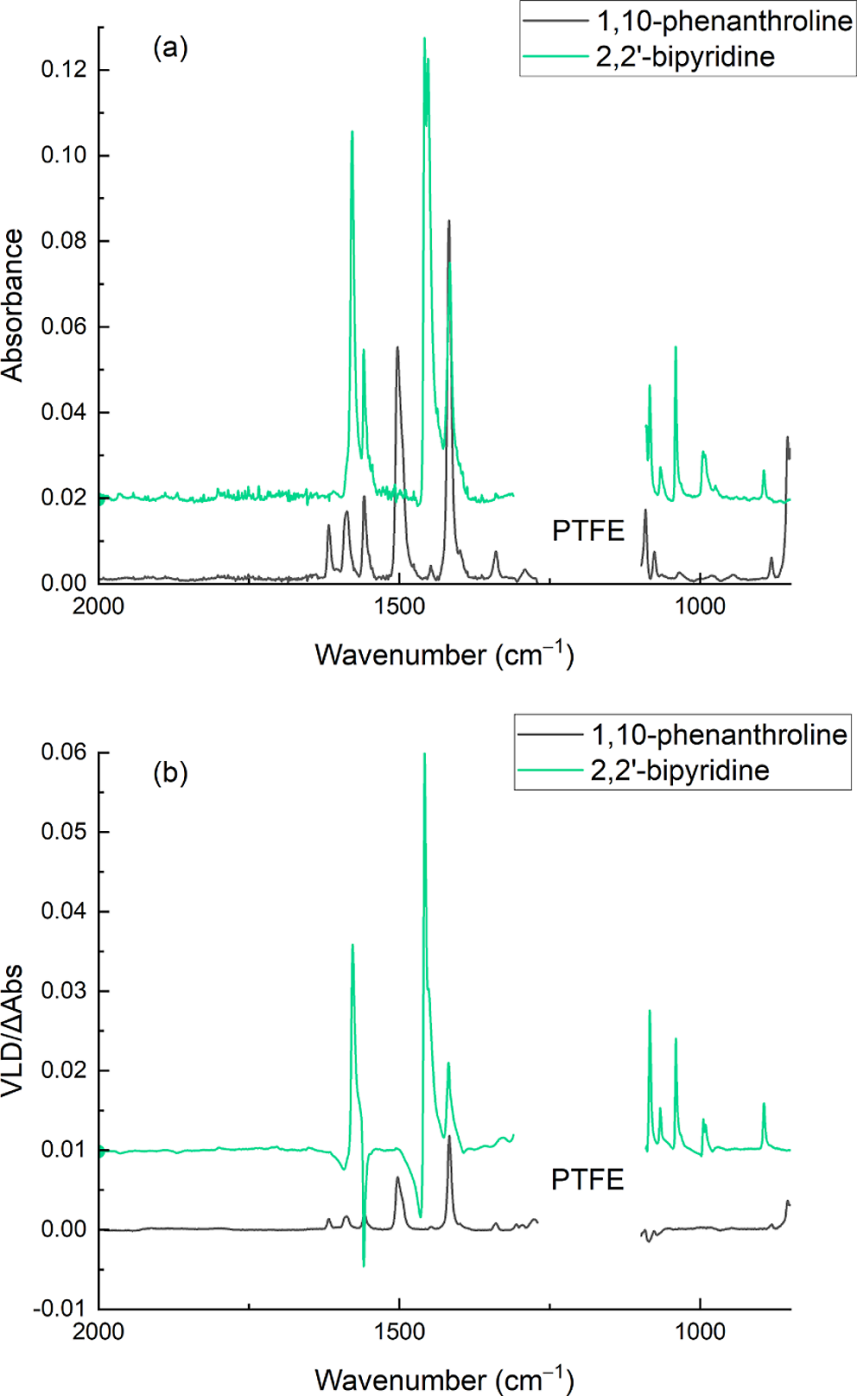
Absorption (a) and VLD (b) spectra of 20 μL of 10 mg/mL 1,10-phenanthroline adsorbed onto the 1.6× stretched PTFE film and 20 μL of 10 mg/mL 2,2′-bipyridine adsorbed onto the 1.89× stretched PTFE film. Polymer spectra have been subtracted, baselines have been flattened, including removal of interference fringes, residual polymer bands have been removed, and the 2,2′-bipyridine spectra are displaced vertically for clarity.

The spectra of 1,10-phenanthroline and 2,2′-bipyridine on stretched PTFE are shown in Figure 7. In this case, the absorbances and VLD values were relatively weak (Abs <0.1 and dAbs <0.01 for phenanthroline) but the good signal-to-noise ratio of the spectrometer ensured that VLD data could be extracted from the spectra of adsorbed molecules with *S* ∼ 0.04. These spectra illustrate the value of having a second polymer that absorbs at different wavenumbers from PE and in this case orients the molecules better (the 1417 cm^–1^ phenanthroline band has LD^r^ = 0.14 on PTFE and 0.050 on PE^OX^). The rate of nitrogen purging has to be controlled for 2,2′-bipyridine to minimize analyte loss during the experiment due to sublimation while minimizing water vapor bands. The exact flow rates for nitrogen depended on the instrument, sample, and variable humidity levels in the laboratory.

**Figure 8:**
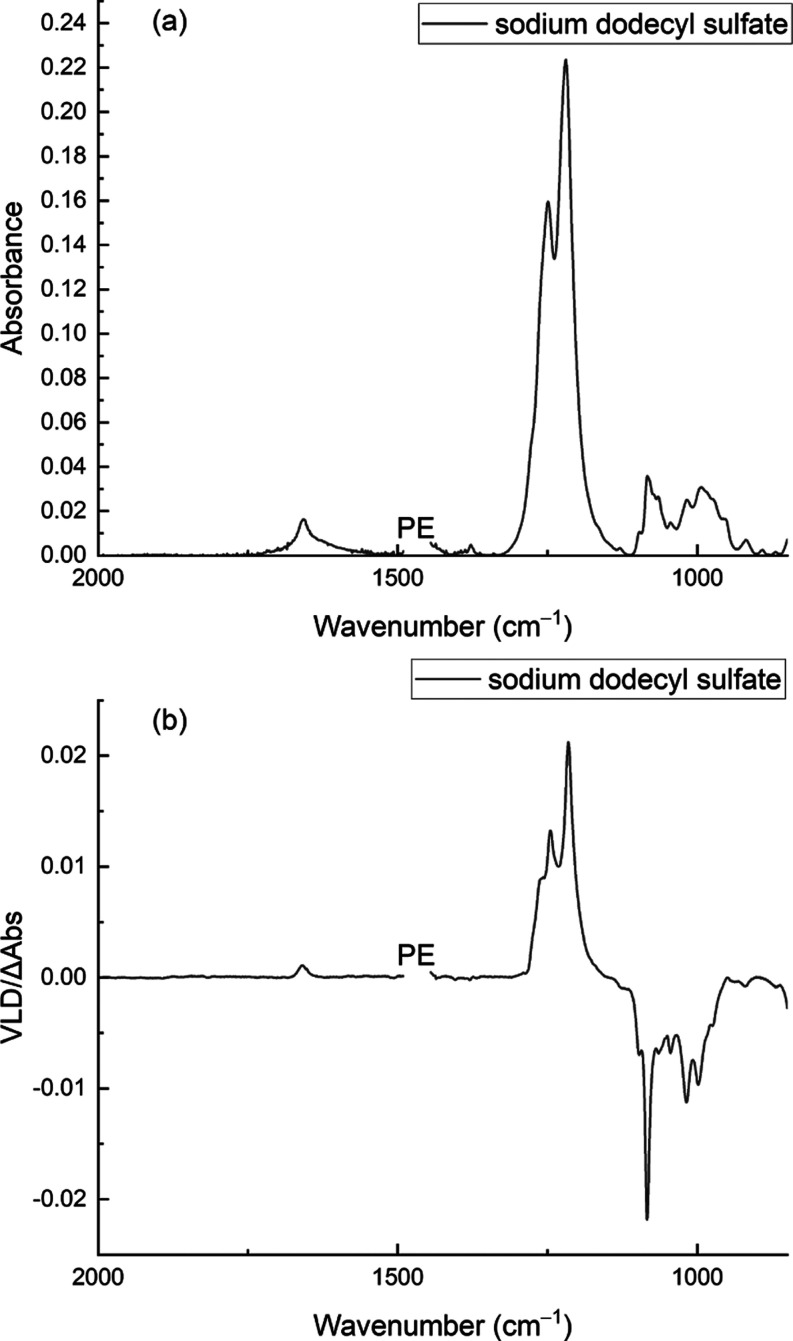
Absorption (a) and VLD (b) spectra of SDS (60 μL of a 1 mg/mL solution in water) adsorbed onto a 2× stretched PE^OX^ film. The polymer background spectrum has been subtracted, the baselines have been flattened, and the residual PE bands have been removed.

The anionic surfactant SDS gives a very good VLD spectrum on PE^OX^ (Figure 8). We are not aware of any other polarized IR spectra of this analyte, although Okabayashi et al.^58^ reported and partly assigned the polarized Raman spectrum below 1200 cm^–1^. We expect the hydrocarbon chains in the dodecyl sulfate ions to align with the stretched PE molecules, thus enabling us to assign transition polarizations. The maximum LD^r^ value measured for the spectrum is −0.61 (*S* = 0.41) for the negative VLD band at 1084 cm^–1^.

### Comparison of Film-Loading Effects in the Spectra for Absorbed and Adsorbed Samples

**Figure 9:**
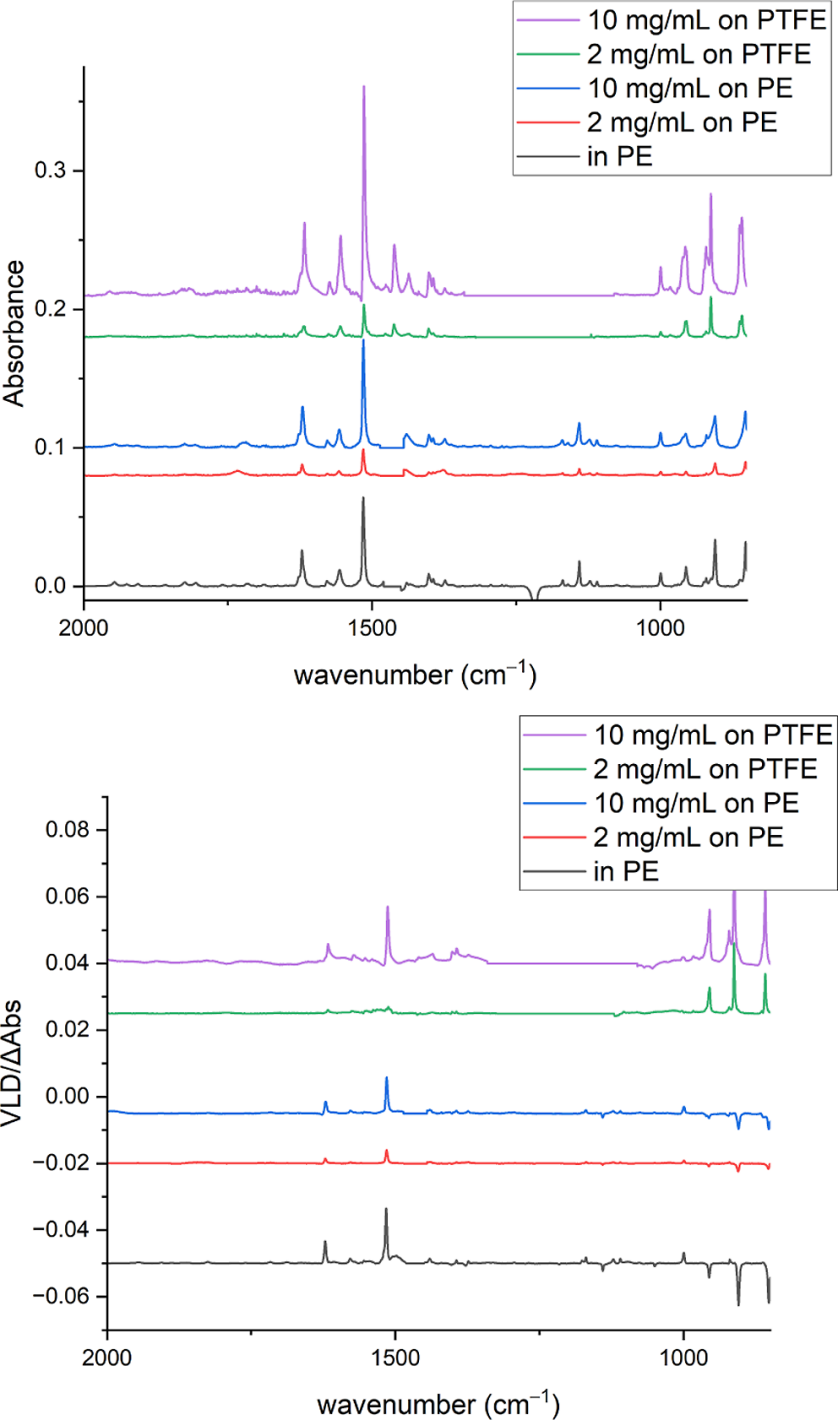
Absorption and VLD spectra of acridine absorbed into 2× stretched Glad Snap-Lock PE; adsorbed onto PE^PnS,OX^; and adsorbed onto 1.9× stretched PTFE. Polymer spectra have been subtracted; baselines flattened; and residual water, PE, and PTFE bands removed. Spectra have been displaced vertically for clarity.

Our goal with our sample presentation approaches for polar (and nonpolar) analytes was to align monodispersed molecules on the surface of the film. As with electronic film LD,^47,59^ the situation may become complicated as the analyte load on the film increases, as illustrated in Figure 9 for acridine adsorbed from chloroform solution (by rapid evaporation) onto PE^OX^ and PTFE films. The lowest spectrum on the graph is of acridine absorbed into 2× stretched Glad Snap-Lock PE^OX^. This is the closest that we have to a reliable polarized spectrum of (presumably) monodisperse acridine. Above this spectrum are two different concentration spectra of acridine adsorbed onto 2× stretched PE^PnS,OX^ and 1.9× stretched PTFE. Overall, the absorption spectra of acridine absorbed into PE and adsorbed onto PE and PTFE films are very similar (subject to different transparency windows) above 1000 cm^–1^, with matrix-induced shifts of 2–3 cm^–1^ for some bands above 1500 cm^–1^. Below 1000 cm^–1^ the absorbed and adsorbed PE LD spectra are consistent; however, the spectrum on PTFE diverges from that of PE^OX^, with multiplets appearing (in both absorption and LD) and the negative bands on PE at 860, 910, and 955 cm^–1^ all becoming positive on PTFE. The high concentration PE absorption spectrum has evidence of the multiplets, though not the LD sign change.

**Figure 10:**
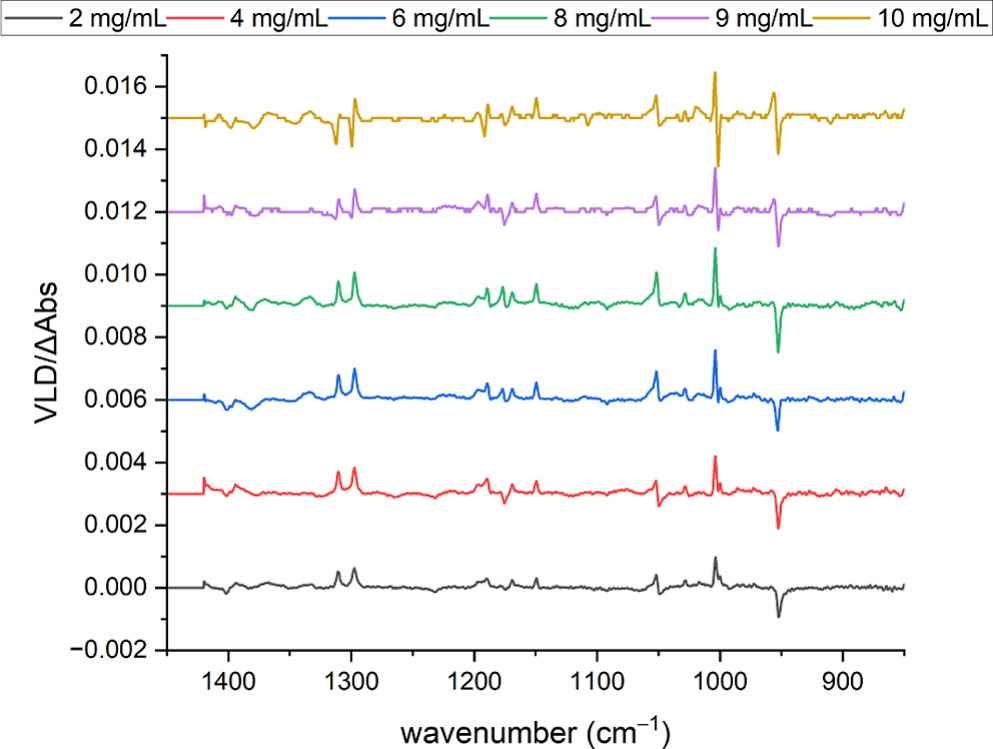
VLD spectra of fluorene adsorbed onto 2× PE^PnS,OX^, produced by drying 20 μL aliquots of 2, 4, 6, 8, 9, and 10 mg/mL fluorene in chloroform on the film. Polymer spectra subtracted; baselines flattened; and residual water and PE bands removed. Spectra have been displaced vertically for clarity.

**Figure 11:**
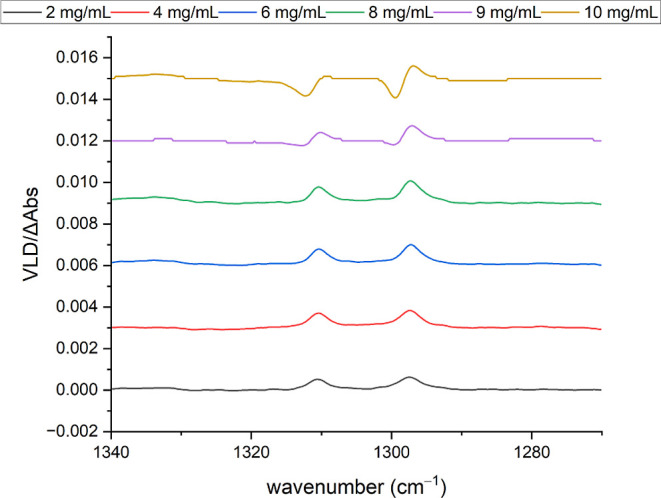
VLD spectra of fluorene adsorbed onto 2× PE^PnS,OX^ produced by drying 20 μL aliquots of 2, 4, 6, 8, 9, and 10 mg/mL fluorene in chloroform on the film. Polymer spectra subtracted; baselines flattened; and residual water and PE bands removed. Spectra have been displaced vertically for clarity.

Equally perplexing features can be observed as a function of the concentration for fluorene on PE^PnS,OX^ (Figures 10 and 11). The low-concentration spectra closely resemble those of fluorene absorbed into PE^OX^ (see Figure 5). Band intensities tend to increase with the sample amount, although variations in sample drying and patch sizes and the tendency for “coffee rings” to form around the patches prevent this from being smooth and quantitative. However, there is a marked change for the higher concentrations, where simple positive or negative VLD bands progressively become sigmoidal in the profile, sometimes leading to a reversal of the VLD sign at higher concentrations. This is also apparent in the fluorene spectra adsorbed onto PTFE and for other analytes such as acridine, anthracene, and 1,10-phenanthroline (see, for example, Figure SI2 in the Supporting Information).

Razmkhah et al.^47^ have shown that the ELD spectra of anthracene adsorbed onto stretched PE^OX^ show evidence of dimerization or more extensive aggregation at higher concentrations, and this may be a factor for some of the analytes that we have studied. However, if dimerization was the dominant mechanism, with the second and possibly subsequent molecules producing slightly shifted absorption bands with opposite LD from the first molecule, then we might expect the unpolarized absorption spectra to become broader, perhaps with some doublet character. This is sometimes, but not always, the case as some combinations of analyte and film give clear evidence of site structure in both the absorption and VLD spectra (see Figure SI2 in the Supporting Information), and the trend toward sigmoidal band profiles for some vibrations at higher surface loadings more closely resembles the features that Miljković et al.,^60^ Schofield et al.,^61^ and other workers reported for IR spectra, notably transflection spectra obtained in IR microspectroscopy. Earlier studies, including ATR spectra, have explained the sigmoidal features in terms of mixtures between the absorption and reflectance spectra. We see them in our transmitted IR spectra for some analyte/polymer systems (see Figure SI2 in the Supporting Information), and typically they are more pronounced in VLD than the absorption spectra. It is likely that this behavior is the result of scattering interfering with the absorption spectrum in regions of rapidly changing refractive index (i.e., absorption regions) when microcrystalline material is present on the surface of the film. This can be minimized by ensuring that the sample is as evenly spread as possible on the film during the drying process and looking at a range of different concentrations; for example, as shown in Figures 10 and 11. Any concentration effects, which could potentially include the formation of multilayers, can be investigated quickly, aided by high sensitivity even at low analyte concentrations.

## Conclusions

Stretched polymer films are valuable sources of electronic and VLD data, and earlier workers obtained good spectra for compounds that can be readily incorporated into a range of available films by measuring independent parallel and perpendicular spectra. In this work, we present a set of vibrational LD spectra collected with a VCD instrument adapted for LD spectroscopy that has enabled us to work with less analyte in the sample and faster data collection. We have also extended the range of stretched polymer films that can be used for nonpolar and polar analytes. We have shown that VLD IR spectra are possible by adsorbing analytes onto commercially available PE in plastic sandwich bags or, for the first time, Glad Press’n Seal film and PTFE tape. These polymer films are commonly available and inexpensive, and samples are more easily prepared than those for many earlier stretched films and crystal studies. Following Razmkhah et al.,^47^ we have used unoxidized and oxidized (PE^OX^) sandwich bags for different polarity analytes. Glad Press’n Seal film for IR spectroscopy proved to have the advantage of a less regular surface, so smaller interference fringes than sandwich bags, and it could also be surface-oxidized for polar analytes. Oxidation changes the polymer very little but creates enough oxygen species on the surface that polar solvents spread uniformly while at the same time having little effect on the PE interaction with nonpolar solvents and analytes. Thus, samples dissolved in any solvent can be deposited on the surface of the PE^OX^ polymer and oriented by the microcrystalline environments of the film. Commercial PTFE tape has proven to be an effective orientation medium with different surface properties and regions of IR transparency from PE. The PTFE tape has previously been used for unpolarized IR spectroscopy,^62^ although not for VLD spectra as far as we are aware.

Data are presented for neutral hydrophobic organic molecules on hydrophobic films including acridine, anthracene, fluorene, and the recently synthesized *S*-(4-((4-cyanophenyl)ethynyl)phenyl)ethanethioate as an example of rapidly obtaining a good-quality VLD spectrum of a new compound. The PE^OX^ films have allowed us to extend our approach to a range of polar or ionic species, including 2,2′-bipyridine, 1,10-phenanthroline and SDS.

In conclusion, notwithstanding the complications that we have observed with some small-molecule analytes, especially at higher sample loadings, the strengths of the combination of direct measurement of VLD spectra and readily available polymer films have enabled us to orient a range of molecules of different polarities and sizes that are too polar and/or too large to penetrate the polymer.

## Supporting Information Available

Synthesis of *S*-(4-((4-cyanophenyl)ethynyl)phenyl)ethanethioate; procedure for baseline correction of IR absorption and VLD spectra; description of IR absorption and VLD spectra of the polymer films used in this work; additional IR absorption and VLD spectra of the polymer films and analytes used in this work; and band wavenumbers, reduced LD (LD^r^), and orientation parameters (*S*) of the VLD spectra of acridine absorbed in ×2 stretched PE films (PDF)
