# Supplementary material for: New Approaches to Stretched Film Sample Alignment and Data Collection for Vibrational Linear Dichroism
Source: ACS Omega. 2023 Sep 28;8(40):37490–500. doi: 10.1021/acsomega.3c05774 (PMC10568702; doi:10.1021/acsomega.3c05774)
Supplement: Supplementary file 1 — ao3c05774_si_001.pdf [file ao3c05774_si_001.pdf]

Supporting Information

New Approaches to Stretched Film Sample Alignment and Data Collection for Vibrational Linear Dichroism

Paul Wormell,*,† Pavel Michal,‡ Adam Scott,§ Koushik Venkatesan,§ Kausala Mylvaganam,§ Tobias von Arx,§ Junya Kitamura,‖ Jun Koshoubu,⊥ and Alison Rodger*,§

† School of Science, Western Sydney University, Locked Bag 1797, Penrith, New South Wales 2751, Australia

‡ Department of Optics, Palacký University Olomouc, 17. Listopadu 12, Olomouc 77146, Czech Republic

§ School of Natural Sciences, Macquarie University, Sydney, New South Wales 2109, Australia

‖ JASCO International Co., Ltd, Hachioji, Tokyo 192-0046, Japan

⊥ JASCO Corporation, Hachioji, Tokyo 192-8537, Japan

* Email p.wormell@westernsydney.edu.au, Phone +61 405 323 631

* Email: alison.rodger@mq.edu.au. Phone: +61 426 627 077

Synthesis of *S*-(4-((4-cyanophenyl)ethynyl)phenyl)ethanethioate

Cyano-4-(ethynyl)benzene (0.50 g, 3.93 mmol), Iodobenzene-4-thioacetate (1.31 g, 4.72 mmol), $Pd(PPh_3)_2Cl_2$ (0.13 g, 0.20 mmol), CuI (0.07 g, 0.39 mmol) and PPh_3 (0.10 g, 0.39 mmol) were suspended in a previously degassed (10 min via N_2 flow) toluene/triethylamine mixture (1:1, 24 mL). The resulting reaction mixture was heated to 40 °C and turned orange within the first 30 min of heating. After 18h of stirring at 40 °C the mixture was allowed to cool to 25 °C and the solvents were evaporated. Purification by column chromatography on silica gel using 4:1 *n*-hexane/ethyl acetate as eluent gave the desired product as an ochre solid (R_f: 0.56, 0.62 g, 2.24 mmol) in 57% yield. ^{1}H NMR (400 MHz, CD_2Cl_2, 298 K) δ 2.43 (s, 3H, CH_3), 7.42-7.43 (m, 1H), 7.44-7.45 (m, 1H), 7.58-7.59 (m, 1H), 7.60-7.61 (m, 1H), 7.63-7.68 (m, 4H). ^{13}C{H} NMR (100 MHz, CD_2Cl_2, 298 K) δ 30.5, 89.5, 92.9, 112.2, 118.8, 123.7, 128.1, 129.9, 132.5, 132.6, 132.7, 134.8, 193.4. IR (ATR-FTIR): ṽ(C=O) 1693.3 cm^{-1}, ṽ(C≡C) 2214.0 cm^{-1}, ṽ(C≡N) 2235.2 cm^{-1}. UV/Vis (CH_2Cl_2, 298 K): λ 305 (48837.2), 323 (44883.7). (+)-HR-ESI-MS (MeOH): calcd for $C_{17}H_{11}NNaOS^+$ $[M + Na]^+$: *m*/*z* 300.0454, found: 300.0451.

Procedure for baseline correction of infrared absorption and VLD spectra

Subtraction of polymer baseline spectra often did not produce analyte spectra with perfectly flat baselines, especially at the sensitivities that were possible using the Jasco FVS-6000 spectrometer. Deviations were attributable to slight mismatches in sample placement and sinusoidal features, discussed below, arising from interference fringes caused by the interaction between the IR beam and the thin polymer films. The Spectra Manager software version 2.07.02 allows spectra to be added, subtracted, and scaled, and for baselines to be corrected by fitting straight lines or splines to different wavenumber segments across the spectrum. Absorbance spectra were routinely flattened in this way when the sharpness of the bands made the location of the baseline clear. The situation was more complicated for broader spectra, including large biomolecules, and correction relied on the comparison of replicate spectra (to account for residual interference fringes) and surface abrasion of the films to minimize the size of the fringes. Other software packages could be used for these purposes, as well as other protocols for baseline flattening.

Due to the baseline correction options only being available for the top channel and the VLD spectra being in the second channel for the Spectra Manager software, the VLD spectra were

flattened by exchanging the absorbance and VLD channels and using the baseline correction function on the VLD spectra, as summarised below. The flattened absorbance and VLD spectra were recombined into a single data file by exporting them to separate Microsoft Excel files, combining the data, and importing the resulting single file back into the Spectra Manager software. Residual PE, PE^{OX} and PTFE bands were removed from the spectra by deleting the relevant absorbance and VLD data points in the Excel files. These regions have been annotated in the Figures to show where the polymer bands have been removed.

The baseline correction software used in this study requires the operator to manually “pin” the correction line (or spline, if this option is chosen) to a series of points on the spectrum, corresponding to points where the location of the baseline is unambiguous. This works best for spectra with little or no undulation due to interference fringes, and sharp bands with good baseline separation. The greater the amount of undulation, the larger the number of “pinned” points that are needed to give a flat baseline between the absorption or VLD bands. See Figure 4 in the main text for an example of baseline correction for a spectrum with sharp spectral features superimposed on an undulating baseline.

Despite careful purging with dry nitrogen, some baseline-corrected spectra contained weak water-vapour rovibrational bands, especially for data collected on humid days, readily identified in the 1 cm^{-1} spectral bandwidth spectra. These were minimized by adding or subtracting small multiples of a standard water-vapour spectrum.

Description of infrared absorption and VLD spectra of the polymer films used in this work

PE and PTFE spectra

When initially mounted in the film stretcher unstretched, and subsequently stretched by an additional factor of two, PE, PE^{OX}, PE^{PnS} and $PE^{PnS,OX}$ films (the longer dimension of the PE^{PnS} roll is the manufacturing stretch direction) gave negative LD bands at 1463 and 1471 cm^{-1}. (Figure SI1) These corresponded to C−H scissoring,[1] consistent with earlier studies.[2] The signs of the bands are consistent with the oriented parts of the PE films having the C−C bonds aligned with the (vertical) stretch direction and C−H bonds perpendicular to it. Smaller positive and negative bands could be observed for other transitions. The LD^r values of the strong negative

bands for the Glad Press'n Seal film are typically about −0.7 indicating that the average orientation parameter of the polymers is $S \sim 0.5$ (assuming $\alpha = 90^{o}$). As Figure SI1 indicates, the Glad Snap Lock PE is less oriented, with $S \sim 0.25$. Regrettably, the PE^{PnS} scatters and/or depolarizes UV-visible light so we could not record corresponding pairs of ELD and VLD spectra using this film.

The PTFE film has absorbance greater than 1.1 for the 1200 cm^{-1} polymer band and hence provides unreliable maximum PTFE LD magnitudes. The PTFE films had typical LD^{r} values of −0.26 (1230 cm^{-1}) and −0.32 (1158 cm^{-1}), corresponding to the C−F bond stretches being perpendicular to the stretch direction, consistent with the spectra of shear-deposited PTFE reported by Ji et al..[3] This gives an average orientation parameter for the polymers of $S \sim 0.1$ (using $\alpha = 90^{o}$).

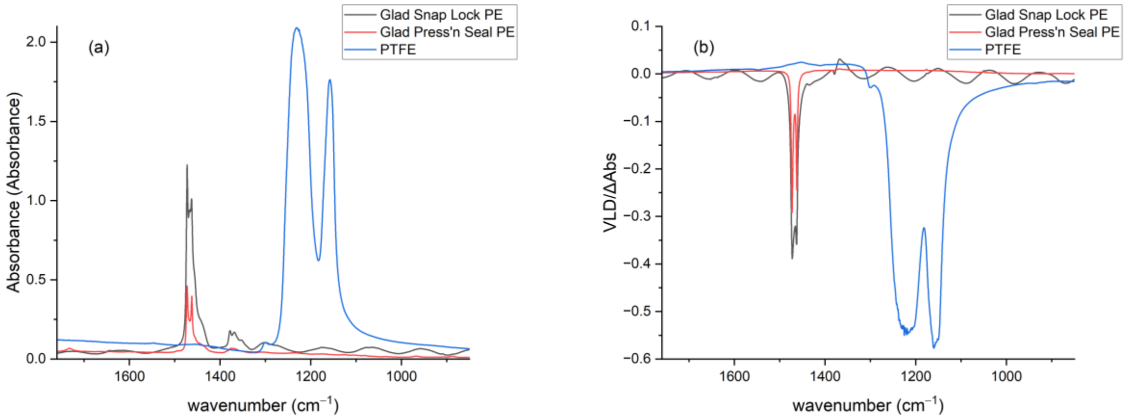

Fig. SI1. Absorption (a) and VLD (b) spectra of 2× stretched Glad Snap Lock PE and $PE^{PnS,OX}$, and 1.55× stretched PTFE films. Baselines have not been flattened, so that interference fringes (PE) and vertical shifts due to scattering are visible.

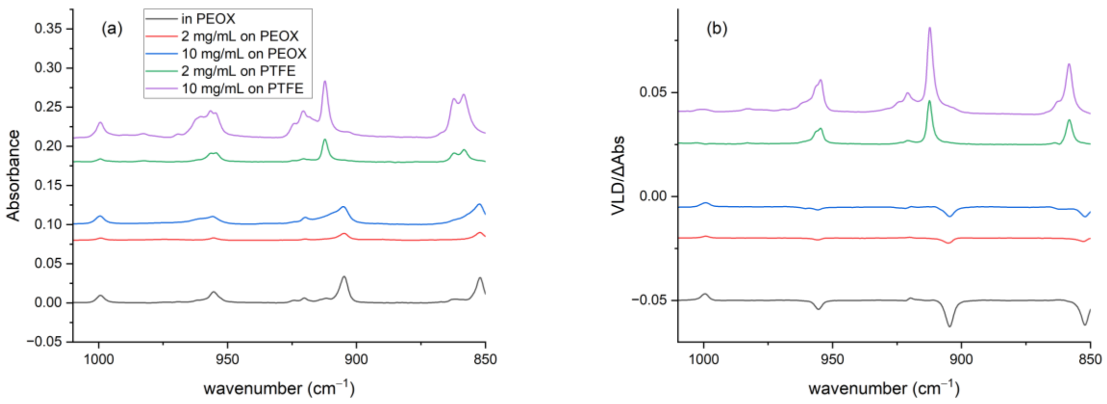

Fig. SI2. Absorption (a) and VLD (b) spectra of acridine absorbed into 2× stretched Glad Snap-Lock PE; adsorbed onto $PE^{PnS,OX}$; and adsorbed onto 1.9× stretched PTFE. Polymer spectra have been subtracted; baselines flattened; and residual water, PE and PTFE bands removed. Spectra have been displaced vertically for clarity.

Table S1. Band wavenumbers, reduced LD (LD^r) and orientation parameters (S) of VLD spectra of acridine absorbed in 2× stretched polyethylene films.

**Literature[5] / cm^{-1}**	**this work Snap-Lock / cm^{-1}**	**Absorbance Snap-Lock**	**LD^r**	***S***		**Absorbance Press'n Seal**	**LD^r**	***S***
**852**(N)	**852**	**0.0263**	**–0.596**	**0.40**		**0.0248**	**–0.181**	**0.12**
	862	0.0010	vw					
905(N)	**905**	**0.0278**	**–0.637**	**0.42**		**0.0218**	**–0.237**	**0.16**
	917	sh						
920(L)	920	0.0076	0.225	0.08		0.0074	0.088	0.03
	924	0.0030	vw					
**956**(N)	**955**	**0.0108**	**–0.555**	**0.37**		**0.0093**	**–0.192**	**0.13**
	962	0.0049	vw			sh		
975(M)	974	vw					vw	
**999**(L)	**999**	**0.0089**	**0.587**	**0.20**		**0.0088**	**0.279**	**0.09**
**1053**(L)	1056	0.0009	vw(+)			0.0009	vw(+)	
1077(L)	1075	0.0013	vw(+)			0.0011	vw(+)	
**1110**(L)	1110	0.0032	0.484	0.16		0.0035	0.263	0.09
**1122**(L)	1122	0.0036	0.397	0.13		0.0039	0.201	0.07
**1140**(M)	**1140**	**0.0141**	**–0.226**	**0.15**		**0.0151**	**–0.153**	**0.10**
	1142	sh						
1160(L)	1160	0.0033	0.121	0.04		0.0022	vw	

Table S1. (continued)

**Literature[5] / cm^{-1}**	**this work Snap-Lock / cm^{-1}**	**Absorbance Snap-Lock**	**LD^r**	***S***		**Absorbance Press'n Seal**	**LD^r**	***S***
1169(L)	1169	0.0046	0.533	0.18		0.0047	0.249	0.06
	1176(VLD)	vw	(+)					
1237(M)	(not seen)							
1267(M)	1268	0.0005	vw(?)			0.0007	vw	
1274(M)	1274	0.0007	vw(–)			0.0011	–0.162	0.11
1293(L)	1294	0.0021	0.297	0.10		0.0013	0.157	0.05
	*c*1300				broad composite			
**1140**(M)	**1140**	**0.0141**	**–0.226**	**0.15**		**0.0151**	**–0.153**	**0.10**
	1312	0.0029	vw(?)			0.0012	vw(?)	
1322(M)						0.0008	vw(?)	
1343(L)	1342	sh	vw(+)		broad composite	0.0005	0.188	0.06
1361(L)						sh.	vw(+?)	
1373(L)	1373	0.0121	?			0.0063	0.132	0.04
	1377	0.0106	–0.186	0.12	composite			
	1378	vw	(–)					
	1387	0.0023	vw(+)					
1394(L)	1394	0.0050	vw(+)			0.0047	0.152	0.05

Table S1. (continued)

**Literature[5] / cm^{-1}**	**this work Snap-Lock / cm^{-1}**	**Absorbance Snap-Lock**	**LD^r**	***S***		**Absorbance Press'n Seal**	**LD^r**	***S***
	1399	sh	(+)					
1402(M)	1401	0.0054	–0.226	0.15		0.0085	–0.099	0.07
	1419	sh	vw(+)					
1431/1440(L)	1437	sh	?		1440 (PnS)	0.0081	0.106	0.04
**PE bands**								
**1516**(L)	**1515**	**0.0621**	**0.313**	**0.10**		**0.0827**	**0.176**	**0.06**
	1555	0.0101	vw(+?)					
1557(M)	1557	0.0101	vw(–?)			0.0138	–0.034	0.02
1578(L)	1577	0.0044	vw(+)			0.0047	0.210	0.07
1588(M)								
	1617	sh	vw(–)					
**1622**(L)	**1621**	**0.0235**	**0.36**	**0.12**		**0.0309**	**0.146**	**0.05**
1627(M)	1627	sh	vw(?)			sh	sh(–)	

Note: Absorbances showed a significant amount of inter-sample variation, depending on sample preparation.

Stronger bands are highlighted in bold text. L = long in-plane axis (Long); M = short in-plane axis (Median); N = out-of-plane axis (Normal)

For L-axis polarized transitions (positive VLD) $S = \frac{LD^r}{3}$. For M- and N-polarized transitions (negative VLD) $S = \frac{-2LD^r}{3}$.

sh denotes shoulder; vw denotes very weak; (+) and (–) denote weak positive and negative LD; and (?) denotes that the observed band is too weak for the sign of the VLD to be discerned.

References

(1) Fontana, L.; Santoro, M.; Bini, R.; Vinh, D. Q.; Scandolo, S. High-pressure vibrational properties of polyethylene. *J. Chem. Phys.* **2010**, *133* (20), 204502.

(2) Krimm, S.; Liang, C. Y.; Sutherland, G. B. B. M. Infrared Spectra of High Polymers. II. Polyethylene. *J. Chem. Phys.* **1956**, *25* (3), 549–562.

(3) Ji, N.; Ostroverkhov, V.; Lagugné-Labarthet, F.; Shen, Y. -R. Surface Vibrational Spectroscopy on Shear-Aligned Poly(tetrafluoroethylene) films. *J. Am. Chem. Soc.* **2003**, *125* (47), 14218–14219.

(4) Farrington, P. J.; Hill, D. J. T.; O'Donnell, J. H.; Pomery, P. J. Suppression of interference fringes in the infrared spectra of thin polymer films. *Appl. Spectrosc.* **1990,** *44* (5), 901–903.

(5) Radziszewski, J. G.; Michl, J. Symmetry assignment of vibrations in anthracene, phenazine, and acridine from infrared dichroism in stretched polyethylene. *J. Chem. Phys.* **1985**, *82* (8), 3527–3533.
